# The ameliorative effect of chrysin on ovarian toxicity caused by methidathion in female rats

**DOI:** 10.3389/fmolb.2024.1470711

**Published:** 2024-11-28

**Authors:** Amany M. Hamed, Fatma Abo Zakaib Ali, Ard elshifa M. E. Mohammed, Muneera Alrasheedi, Islam Ragab, Maryam Aldoghaim, Safaa S. Soliman

**Affiliations:** ^1^ Chemistry Department, Faculty of Science, Sohag University, Sohag, Egypt; ^2^ Department of Pathology and Clinical Pathology, Faculty of Veterinary Medicine, Sohag University, Sohag, Egypt; ^3^ Department of Chemistry, College of Science, Qassim University, Buraidah, Saudi Arabia; ^4^ Department of Chemistry, College of Science, King Faisal University, Al-Ahsa, Saudi Arabia; ^5^ Department of Zoology, Faculty of Science, Minia University, Minia, Egypt

**Keywords:** methidathion, chrysin, inflammation, ovarian damage, oxidative stress

## Abstract

**Introduction:** Methidathion (MD) is commonly used in agriculture and has adverse effects on reproduction. Chrysin (CHR) has several advantageous properties, such as anti-inflammatory, anti-cancer, and antioxidant properties. The purpose of the current investigation was to assess CHR’s therapeutic efficacy in reducing ovarian toxicity brought on by MD.

**Methods:** Twenty-four female rats were divided into four groups of six animals each. Group 1 served as a control, while group 2 rats received MD (5 mg/kg). Rats in Group 3 received CHR at a dose of 50 mg/kg. Rats in group 4 received treatment with CHR after MD intoxication.

**Results and Discussion:** Our research revealed that MD significantly (*p* < 0.001) increased the levels of MDA, caspase-3, FSH, LH, CA-125, and TNF-α but significantly (*p* < 0.001) decreased the levels of SOD, GSH, E2, and progesterone when compared to the control and CHR groups. After receiving CHR therapy, damage induced by MD was significantly (*p* < 0.001) repaired.

**Conclusion:** This study showed that CHR could mitigate the adverse effects that MD causes to the ovaries by decreasing oxidative stress, inflammation, and apoptosis; improving antioxidant status; and restoring the correct ratio of sex hormones.

## Graphical Abstract

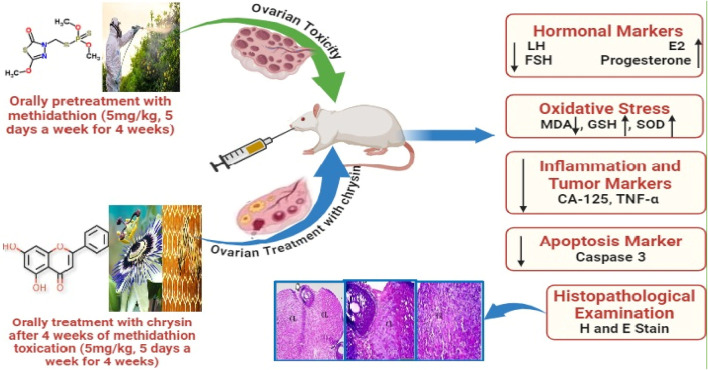

## 1 Introduction

Since organophosphorous pesticides (OPPs) are used for eliminating insects that harm crops in the field, they fall under the category of insecticides. Nevertheless, other data indicates that these substances may also be employed as herbicides by influencing the soil biota population and subsequently changing the biomass of plants, leading to their deterioration (Ajiboye et al., 2022). OPPs, one of the main chemicals used to control pests, have been widely utilized in agriculture since the ban on organochlorine pesticides (Costa, 2018). Acetylcholinesterase (AChE), a vital and essential enzyme to produce nerve impulses, is inhibited by the chemical organophosphate. Acetylcholine accumulates as a result of AChE inhibition, permanently depolarizing the organism and causing tremors, respiratory arrest, and eventually death. Owing to the widespread application of these chemicals on agricultural products, they have infiltrated groundwater through seepage, entered rivers via agricultural runoff, and appeared on the surfaces of sprayed plants. Thus, food, inhalation, and skin adsorption have been the primary routes by which people have been exposed to lower concentrations of these pesticides (Costa, 2018; González-Alzaga et al., 2014). There are 220,000 deaths and one million cases of severe poisoning each year; most of these poisonings and 99% of the deaths they cause happen in developing countries (Abdulrahman et al., 2023).

OPPs toxicological effects are classified as mild, moderate, or high depending on when the person is exposed to them and what proportion of their enzymes are inhibited afterward. Due to the rising levels of pesticides in the environment, established and developing nations that rely heavily on agriculture for their economies now have virtually no choice but to expose living things to them (Georgiadis et al., 2018). OPPs have been demonstrated to trigger biochemical and histological alterations in various organs, including the kidney, immune system, pancreas, liver, heart, and vascular walls. The ovary is one of the organs discussed, and it has a crucial function by generating oocytes and synthesizing hormones in a normal reproductive process (Güney et al., 2007). Moreover, additional studies indicate that oxidative stress can play a major role in the toxicity mechanism of OPPs. These pesticides can cause oxidative stress, which can result in the generation of free radicals and changes to antioxidants or the enzymes that scavenge reactive oxygen species (ROS) (Sharma et al., 2014), which coexist in a healthy body and are balanced with antioxidants. The process that results in an imbalance that generates an excess of ROS is known as oxidative stress. A woman is affected by oxidative stress throughout her whole reproductive life, even after menopause. An imbalance between the body’s ability to scavenge free radical species and antioxidants results in oxidative stress. In addition to being crucial signaling molecules in physiological processes, ROS are involved in pathological processes that impact the female reproductive system. ROS influences a wide range of physiological processes, including oocyte maturation, fertilization, embryo development, and pregnancy. Both DNA damage to the ovarian epithelium and oxidative base damage caused by ovulation can be prevented by antioxidants (Agarwal et al., 2005).

Methidathion [*S*-(5-methoxy-2-oxo-2,3-dihydro-1,3,4-thiadiazol-3-yl)methyl *O*,*O*-dimethyl phosphorodithioate] (Kim et al., 2011) is one of the OPPs that is most frequently employed in public health and agriculture initiatives (Bhattu et al., 2022).

A flavonoid called chrysin (5, 7-dihydroxyflavone) is taken from propolis, passion flowers, and honey (Naz et al., 2019). Chrysin is used to treat liver, nervous system, and reproductive disorders (Mentese et al., 2022; Souza et al., 2015). Numerous biological characteristics of chrysin, such as its antioxidant, anti-apoptotic, anti-inflammatory, and anti-cancer capabilities, have been demonstrated by previous studies (He et al., 2012; Rashno et al., 2019). Moreover, the protective benefits of chrysin against oxidative stress in rats have been determined. According to the study, chrysin therapy lowers MDA levels and increases antioxidant enzyme activity (Anand et al., 2012). Additionally, it has been demonstrated to regulate the level of sexual hormones, protect ovarian tissues from oxidative stress and tissue damage, and reduce apoptosis (Mohammadi et al., 2022). Therefore, this study aimed to investigate the ameliorative effect of chrysin against MD-induced ovarian damage in female rats by evaluating serum biochemical antioxidative markers, sex hormones, and inflammatory and apoptotic markers, in addition to histopathological examinations of ovarian tissue.

## 2 Materials and methods

### 2.1 Chemicals

Methidathion (CAS No. 950-37-8), Chrysin (CAS No. 480-40-0), and other reagents were supplied by Sigma-Aldrich (St. Louis, MO, United States). The selection of MD and CHR doses was based on previously published studies by Sulak et al. (2005) and Mantawy et al. (2017) respectively.

### 2.2 Ethical considerations

The housing accommodations and experimental protocols followed the European Union Council’s (2010/63/EU) guidelines for the use of laboratory animals. The animal study and all experimental procedures have been approved by the veterinary medical research ethics committee, Faculty of Veterinary Medicine, Sohag University, Sohag, Egypt, with protocol number Soh. un.vet/00066R. Every precaution was taken to prevent pain and suffering for the animals.

### 2.3 Animals

Female Wistar albino rats, weighing between 180 and 200 g and aged 10–12 weeks, were obtained from the animal house at Sohag University in Sohag, Egypt. The rats were maintained at a constant temperature of 24°C ± 1°C with a 12-hour light/dark cycle and 45% ± 5% humidity. They were acclimated to their new environment for 1 week before the experiment. Throughout the study, water and food were provided *ad libitum*.

### 2.4 Experimental design

Animals were randomly divided into four groups (6 rats/group) as follows: Group 1 (vehicle control): healthy rats that received DMSO orally only, Group 2 (MD): ovarian toxicity was induced by methidathion (5 mg/kg.b.w), Group 3 (CHR): healthy rats that received chrysin (50 mg/kg), and Group 4 (MD + CHR): ovarian toxicity was induced by MD the same as in group 2, then rats received chrysin at the same dose mentioned in group 3. Treatment with CHR started 4 weeks after ovarian toxicity induction by gavage 5 days a week and continued for 5 days a week for 4 weeks by gavage administration. MD and CHR were dissolved in DMSO, and doses of MD and CHR were determined from previous studies of Sulak et al. (2005), Mentese et al. (2022), Mantawy et al. (2017). At the end of the treatment, animals were sacrificed and dissected.

### 2.5 Blood sampling for hormonal and biochemical analyses

Blood samples were collected and centrifuged for 10 min at 3,000 rpm to obtain a clear serum and stored at −20°C for hormonal and biochemical analyses. Ovarian tissue samples were dissected and processed for biochemical analysis, and histopathological examination.

### 2.6 Determination of sex hormone

Rats’ serum levels of progesterone, estrogen (E2), follicular stimulating hormone (FSH), and luteinizing hormone (LH) were quantitatively assessed by ELISA using kits [PerkinElmer Company, Hayward, CA 94545 for progesterone (Catalogue Number: 10005), and BIOS Company, South San Francisco, CA 94080, United States for LH (Catalogue Number: 1004), FSH (Catalogue Number: 10001), and (E2 (Catalogue Number:1009)]. The experiment was carried out according to the manufacturer’s guidelines.

### 2.7 Determination of oxidative stress

All the rats’ ovaries were removed immediately and weighed. In a glass homogenizer, each rat’s right ovary was homogenized in cold phosphate-buffered saline (1:4) (pH 7, 0.01 mol/L). The homogenate that was created was centrifuged at 5,000×g for 5 min, filtered, and utilized to measure markers of oxidative stress. The experiments were repeated three times.

#### 2.7.1 Determination of SOD

The body’s antioxidant capacity and oxidative balance can be determined by measuring the activity of SOD, an enzyme that scavenges superoxide anion-free liquid (O2 - •) to protect cells from damage (Liang et al., 2022). The total SOD assay kit (MyBioSource, China, Catalogue Number: MBS036924) was used in this experiment to measure SOD activity (U/mg protein) in ovarian homogenates of each group, according to the instructions.

#### 2.7.2 Determination of MDA

Through the action of its enzyme system, which can target polyunsaturated fats in biological membranes, initiate lipid peroxidation, harm cells, and generate lipid peroxides like MDA, the body produces oxygen-free fluid. As a result, the body’s measured MDA concentration may indicate the level of lipid peroxidation and, in consequence, indicate the degree of cell damage (Yang et al., 2022). Using the TBA method MDA detection kit (MyBioSource, China, Catalogue Number: MBS268427), the researchers measured the amount of MDA in each group’s ovarian tissue. Following the instructions provided in the kit, the ovarian tissues were homogenized and the concentration of MDA per mg protein (nmol/mg protein) was determined.

#### 2.7.3 Determination of GSH

GSH is an essential enzyme that is extensively distributed throughout the body and catalyzes the breakdown of H2O2. Its action may contribute to maintaining the cell membrane’s structural and functional integrity. Consequently, assessing the GSH activity in tissues may provide information regarding the body’s oxidative equilibrium and antioxidant potential (Yang et al., 2022). In this work, the reduced GSH activity in ovarian homogenates of each group was measured according to the instructions using a GSH test kit (Shanghai BlueGene Biotech Co., Ltd., Shanghai, China, Catalogue Number: E02G0367).

### 2.8 Detection of ovarian apoptosis

One important protease in the pathway of mitochondria-mediated cell death is caspase 3. This factor is released into the cytoplasm in response to apoptotic stimuli, where it sets off a series of processes that lead to cell death. Using an ELISA kit (United States of America, from Elbscience Biotechnology Company, Catalogue Number: E-EL-R0160), we determined the concentration of Caspase3 in each group’s serum by the manufacturer’s instructions.

### 2.9 Detection of tumor marker

A significant percentage of epithelial ovarian tumors exhibit the high molecular weight glycoprotein known as carcinoembryonic antigen (CA) 125 (Moss et al., 2005). Serum level was quantitatively assessed by ELISA using a kit from BIOS Company, South San Francisco, CA 94080, United States of America, Catalogue Number: 10,103. The experiment was carried out according to the manufacturer’s guidelines.

### 2.10 Detection of cytokine

The oocytes and macrophages of the neonatal rat ovary contain the multifunctional cytokine known as tumor necrosis factor α (TNFα). TNFα may play a role in follicle construction or oocyte atresia, as evidenced by the presence of both the TNFα and its receptors in the ovary of a newborn rat (Morrison and Marcinkiewicz, 2002). The serum level was quantitatively assessed by ELISA using a kit from R&D Systems in Minneapolis, MS, United States of America, Catalog Number QK210. The experiment was carried out by the manufacturer’s guidelines.

### 2.11 Histopathological examination

Animals were sacrificed after the experimental duration, and tissue samples from left ovaries were collected, dissected, and immediately fixed in 10% formalin for 24 h, dehydrated in a succession of graded alcohols, clarified in xylene, and encapsulated in paraffin (Suvarna and Layton, 2013). Tissue sectioning was done at 3–5 μm thickness and stained with hematoxylin and eosin (H&E) (Bancroft et al., 1996) for histological evaluation. All sections were inspected and photographed using an OLYMPUS CX43 microscope and a microscope-adapted OLYMPUS SC52 camera.

### 2.12 Morphometric study

Each animal was assigned a score based on histopathological examination of the tissue samples (Gibson-Corley et al., 2013). The section samples were scored semi-quantitatively, depending on the visual field inspection of 10 sections from each group. Photographs of ovarian tissues were taken, and the cellular alterations were counted in 10 random areas (each 1 mm^2^) at a magnification of 20×. The degree of follicular degeneration, stromal degeneration, and stromal fibrosis was scored between 0 and 3 according to the severity of the damage. A value of 0 means no pathological damage, +1 value is less than 33% of ovarian damage, +2 is damage between 33% and 66%, and +3 is more than 66% (Pala et al., 2014; Kaplan et al., 2021).

### 2.13 Statistical analysis

The Statistical Package for the Social Science (S.P.S.S. version 27) was used to analyze the data. The mean ± SD was used to express the results. To test differences between groups, statistical analysis was performed using analysis of one-way variance (ANOVA) and Tukey’s post-hock multiple comparison test. Each group’s results were characterized by identical variance and a normal distribution. A value of *p* of <0.05 means that differences between all groups are statistically significant.

## 3 Results

### 3.1 Clinical observation

Following MD administration, the rats displayed intense, uncontrolled behaviors that lasted for nearly 2 hours, after which they showed signs of exhaustion. In the subsequent days, the severity of these behaviors persisted but gradually diminished. No mortality was observed in any group.

### 3.2 Biochemical findings

#### 3.2.1 Chrysin regulate serum hormonal markers in methidathion-treated rats

**FIGURE 1:**
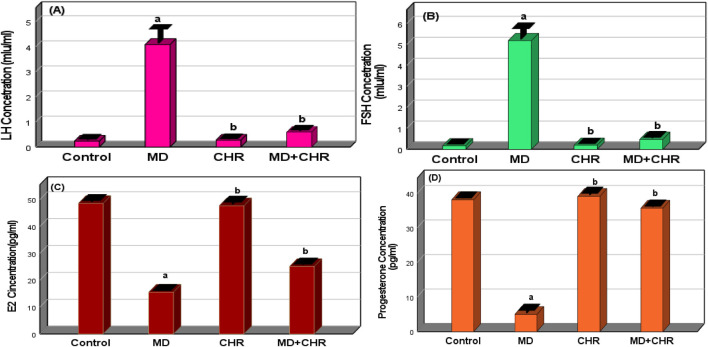
Effect of CHR on the serum hormonal markers in experimental groups. **(A)** LH concentration, **(B)** FSH concentration, **(C)** Estradiol concentration, **(D)** Progesterone concentration. Data expressed as mean ± SD, ^a^p < 0.001 versus the control group; ^b^p < 0.001 versus MD group, (n = 6).

The hormone values of experimental groups are displayed in (Figure 1). In the current investigation, the FSH and LH hormone serum levels were considerably higher (*p* < 0.001) in the MD group than in the control and CHR groups. There was no significant difference (*p* > 0.05) in the serum levels of LH and FSH hormones between the CHR and control groups. In contrast to the MD group, the LH and FSH hormone levels were considerably lower in the MD + CHR treated group (*p* < 0.001). (Figures 1A, B).

Comparing the MD group to the control and CHR groups, the MD group’s serum levels of progesterone hormone and E2 were significantly lower (*p* < 0.001). Progesterone hormone and E2 levels in the MD + CHR group’s blood increased significantly (*p* < 0.001) following CHR treatment. There was no discernible difference in progesterone hormone and E2 serum levels between the CHR and control groups. (Figures 1C, D).

#### 3.2.2 Chrysin improved ovarian tissue antioxidants and oxidative stress markers in methidathion-treated rats

When comparing the MD untreated group to the control group, (Table 1), the levels of MDA were substantially elevated (*p* < 0.001) along with a significant decline in GSH level and SOD activity. Surprisingly, the CHR treatments improved GSH and SOD activity and dramatically downregulated (*p* < 0.001), MDA levels in the MD + CHR group in comparison with the MD group. These findings indicated that CHR mitigated the oxidative stress induced by MD treatment in the rats that received it.

**TABLE 1:** Effect of CHR on the ovarian tissue antioxidants and oxidative stress markers in experimental groups.

Groups	MDA nmol/mg	GSH pg/g	SOD U/mg
Control	0.34 ± 0.05	2.79 ± 0.03	6.19 ± 0.05
MD	5.05 ± 0.08^a^	0.48 ± 0.03^a^	2.20 ± 0.16^a^
CHR	1.14 ± 0.09^b^	3.55 ± 0.04^b^	7.38 ± 0.09^b^
MD + CHR	0.80 ± 0.01^b^	2.19 ± 0.034^b^	5.64 ± 0.06^b^

Values are presented as mean ± SD; n = 6 rats in each group; values with different superscripts (a, b) among experimental groups are significantly different (*p* < 0.001).

^a^ Versus the control group.

^b^ Versus MD, group using ANOVA, and *post hoc* test; SD, standard deviation.

#### 3.2.3 Effect of chrysin on the serum ovarian inflammation and tumor markers in methidathion-treated rats

The levels of TNF-α and CA-125 in the MD rats were greatly higher (*p* < 0.001) than those in the control rats. However, after CHR treatment, the values in the MD + CHR group were notably lower (*p* < 0.001). Additionally, there was no significant difference (*p* > 0.05) in the levels of TNF-α and CA-125 between the control and CHR groups. These findings suggest a strong association between the cytokine system and the tumor marker CA-125. (Table 2). Consequently, it can be concluded that CHR treatment effectively reduces the inflammation caused by MD treatment in rats.

**TABLE 2:** Effect of CHR on the ovarian inflammation and tumor markers in experimental groups.

Groups	CA-125 (U/mL)	TNF-α (pg/mL)
Control	22.35 ± 0.68	52.30 ± 0.38
MD	54.32 ± 0.57^a^	234.79 ± 0.78^a^
CHR	30.54 ± 0.63^b^	61.83 ± 0.61^b^
MD + CHR	27.28 ± 0.33^b^	49.63 ± 0.51^b^

Values are presented as mean ± SD; n = 6 rats in each group; values with different superscripts (a, b) among experimental groups are significantly different (*p* < 0.001).

^a^ Versus the control group.

^b^ Versus MD, group using ANOVA, and *post hoc* test; SD, standard deviation.

#### 3.2.4 Effect of chrysin on the serum caspase 3 (Apoptosis Marker) of methidathion-treated rats

**FIGURE 2:**
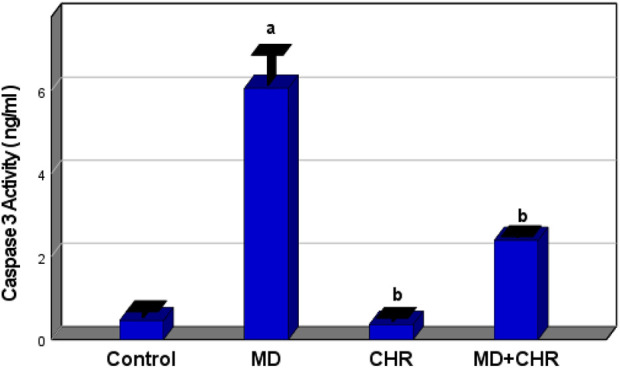
Effect of CHR on the serum caspase3 (Apoptosis Marker) in experimental groups. Data expressed as mean ± SD, ^a^p < 0.001 *versus* the control group; ^b^p < 0.001 *versus* MD group, (n = 6).

There was a significant increase (*p* < 0.001) in the serum Caspase-3 activity level in the MD group compared to the control group. In contrast, the MD + CHR group receiving CHR therapy saw a significant drop in the mean value of Caspase-3 activity. Serum Caspase-3 levels showed no noticeable variations between the control and CHR groups. (Figure 2).

### 3.3 Histopathological assessment

**FIGURE 3:**
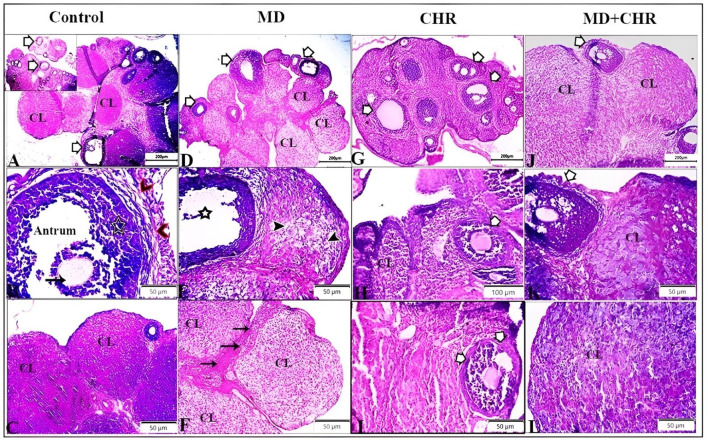
Representative photomicrographs of ovarian tissue samples from treated groups stained by hematoxylin and eosin **(H, E)** demonstrate the control group: **(A)**: different stages of ovarian follicles (arrows), and corpus luteum (CL). **(B)**: Graafian (secondary) follicle with a large follicular antrum. Secondary oocyte located eccentrically and surrounded by the zona pellucida and a layer of several cells known as the corona radiata (arrow), surrounded by multiple cellular layers of granulosa cells (star), and theca cell layers (arrowheads). **(C):** normal size and structure of corpus luteum (CL). MD group **(D–F)**: **(D)**: Number of atretic follicles with degenerated follicular cells (arrows), degenerated corpora bodies (CL). **(E):** Abnormal secondary follicles without oocyte (star), Corpus luteum showing deteriorating luteal cells with vacuolation (arrowheads). **(F)**: Corpora showed degenerative changes marked by loss of cellular features and cellular vacuolation (CL) with increased inter-corpora stromal fibrous connective tissue (arrows). CHR group **(G–I)**: Number of normal ovarian follicles (arrows), and corpus luteum (CL). MD + CHR group **(J-L)**: the presence of growing healthy follicles containing an oocyte with an intact zona pellucida and surrounded normal granulosa cells (arrows), more or less corpus luteum containing polyhedral luteinized cells (CL).

The histological examination of ovarian tissue samples revealed distinctive morphological differences between the treated groups and the control as shown in (Figure 3). In the control group, normal ovarian tissue exhibited various stages of ovarian follicles, including a Graafian (secondary) follicle with a well-defined follicular antrum housing an eccentrically positioned secondary oocyte, surrounded by the zona pellucida and corona radiata. This follicle was enveloped by layers of granulosa and theca cells. Furthermore, the corpus luteum maintained a typical size and structure (Figures 3A–C). Contrarily, the MD group displayed numerous atretic follicles characterized by degenerated follicular cells and compromised corpora bodies. Aberrant secondary follicles lacking oocytes were observed alongside the corpus luteum exhibiting luteal cell vacuolation. Degenerative changes, including cellular loss of features and vacuolation, were evident in the corpora, accompanied by an increase in inter-corpora stromal fibrous connective tissue (Figures 3D–F).

In comparison, the CHR group showed a comparable number of normal ovarian follicles and corpus luteum structures to the control (Figures 3G–I). Remarkably, the MD + CHR group exhibited a notable improvement in ovarian structures. Healthy growing follicles containing intact oocytes surrounded by normal granulosa cells were observed, alongside more or less normal corpus luteum structures with polyhedral luteinized cells (Figures 3J–L).

**FIGURE 4:**
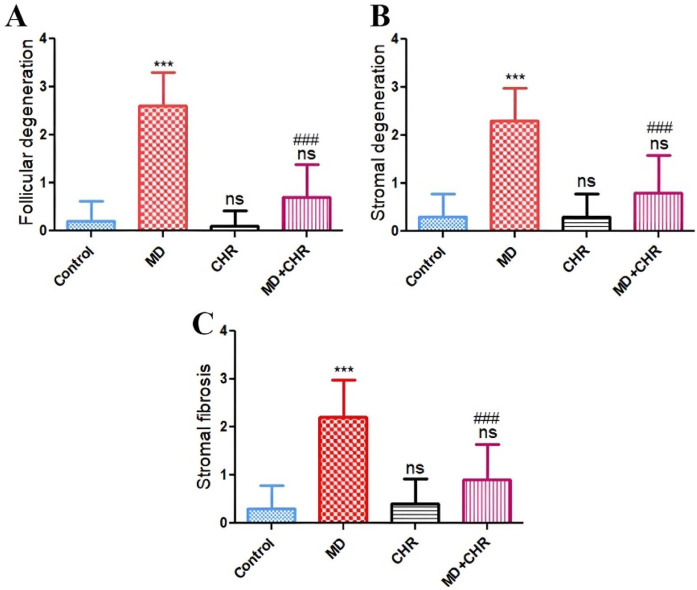
Histomorphometry graph showing semiquantitative measurements of lesion scores recorded in ovarian tissue sections among the experimental groups **(A):** Follicular degeneration, **(B):** Stromal degeneration, and **(C):** Stromal fibrosis. Results were analyzed using one-way ANOVA and Tukey’s *post hoc* tests. Results are shown in mean ± SD. ^***, ###^ *p* ≤ 0.001 compared to control, and MD groups respectively. ns = *p* > 0.05 (non-significant vs. Control).

Histomorphometric evaluation of ovarian lesions such as follicular degeneration, stromal degeneration, and stromal fibrosis recorded in the examined tissue sections exhibits a significant (*p* ≤ 0.001) increase in the MD group compared with the control group. Interestingly administration of CHR dramatically decreased those recorded lesions (*p* ≤ 0.001) compared with the MD untreated group. The histological picture in MD + CHR showed non-significant change compared with the control group (Figure 4).

## 4 Discussion

The present study investigated the therapeutic potential of CHR against MD-induced ovarian damage in female rats.

Concerning hormonal balance, the MD group in this study had considerably higher serum levels of FSH and LH and significantly lower serum levels of progesterone and E2. Severe toxicity to the follicular ovarian reserve and function is another explanation for this effect. The presence of these hormonal alterations is utilized as a sign of ovarian failure (Afifi and Reyad, 2013). Our findings match up with a previous study (Güney et al., 2007), which reported that MD induced ovarian toxicity in female rats. Additionally, compared to the MD group, the group treated with CHR after intoxication with MD showed a significant decrease in FSH and LH levels along with a significant increase in estradiol and progesterone levels. The beneficial effects of CHR on the reproductive processes of female rats may have contributed to our findings (Mentese et al., 2022). Our findings are consistent with those of Mentese et al. (2022), who displayed that CHR prevention female rats from ovarian toxicity.

Additionally, our results suggested that administering MD to normal rats resulted in a substantial drop in GSH and SOD levels that were comparable to those of normal rats as well as a significant increase in MDA levels. This may be because MD poisoning causes a variety of metabolic disturbances (Kose et al., 2009). Oxidative stress is caused by antioxidant enzyme deactivation (Mobasher and Valverde, 2014). This finding is consistent with the results of Wang et al. (2018), which associated increased MDA levels and decreased GSH and SOD levels with acrolein’s interference in antioxidant defense mechanisms. Oxidative stress can lead to ovarian failure by impairing CYP450 in two ways: disrupting normal oocyte development and inducing apoptosis. Since oxidative stress hinders both nuclear and cytoplasmic maturation of oocytes while promoting cell death, it disturbs the intraovarian environment by creating an imbalance between ROS production and elimination. In the present study, elevated ovarian MDA levels may be linked to increased reactive oxygen species induced by MD (Güney et al., 2007). These observations support the hypothesis that MD-induced ovarian damage results from lipid peroxidation (LPO), a biochemical mechanism. Developing effective antidotes, particularly those with strong antioxidative properties, is crucial to counteracting MD toxicity. Chrysin, a flavonoid with potent antioxidant activity, represents a promising candidate in this regard (Alsawaf et al., 2022).

According to the author’s knowledge, there is limited information about the potential benefits of CHR in combating MD toxicity. Chrysin is a potential naturally occurring flavonoid that is usually found in propolis and honey. Because of its anti-inflammatory and antioxidative features, it has a protective effect (Farkhondeh et al., 2019). Chrysin’s mode of action involves reducing inflammation, inducing apoptosis in cells, and decreasing cell proliferation without harming healthy cells or having any unfavorable side effects (Xue et al., 2016; Kasala et al., 2015; Samarghandian et al., 2011). In comparison to the MD group, treating MD rats with CHR resulted in significant increases in ovarian levels of GSH and SOD and significant decreases in ovarian MDA levels. These results are consistent with those of Ye et al. (2022), who discovered that rats receiving CHR therapy showed a significant return of these parameters to normal. Additionally, CHR demonstrated improved antioxidant status, a decrease in oxidative stress, and the prevention of the generation of free radicals (Temel et al., 2020). It has been found that phytocompounds, especially flavonoids, can shield biological macromolecules and membranes against damage caused by free radicals (Karak, 2019). Consistent with these outcomes, the current research demonstrated that flavonoid compounds found in CHR may reduce the oxidative stress that MD causes in MD rats by increasing antioxidant status and lowering lipid peroxidation levels (Alsawaf et al., 2022). According to earlier research, the compound’s antioxidant qualities and ability to eliminate free radicals are assumed to be attributed to the hydroxyl groups at positions 5 and 7 in the CHR structures (Eldutar et al., 2017). CHR therapy has been shown by some other authors (Rehman et al., 2013) to enhance antioxidant enzyme activity and protect tissues from oxidative damage.

The cytokine TNF-α was first discovered to play a part in inflammatory processes. This factor activates by attaching itself to one of its two receptors, a type 1 receptor (TNFR1) and a type 2 receptor (TNFR2). TNF-α stimulates CA-125 in breast, endometrial, and ovarian cancers through nuclear factor kappa B (NF-κB) (Morgado et al., 2016). Moreover, TNF-α plays a role in controlling physiological processes like corpus luteum function, steroidogenesis, ovulation, and follicular growth. Additionally, it has been documented that TNF-α may control granulosa cell differentiation and death according to the embryonic stage (Silva et al., 2020). According to certain research, serum TNF-α receptor 1 binds to CA-125 more frequently than receptor 2 (Rzymski, 2005). TNF has been linked to elevated serum CA 125 levels, according to a prior study. These findings imply that cytokines and CA-125 might be related (Kosar et al., 2006). Furthermore, ovarian follicular loss is significantly impacted by the inflammatory response (Mantawy et al., 2019). An increasing number of research has shown that abnormal inflammation can change the dynamics of the ovarian follicles in a way that can lead to infertility (Boots and Jungheim, 2015; Urieli-Shoval et al., 2013). In rats exposed to Organophosphate-Pesticide, serum level of TNF-α was increased (Alam et al., 2019). Our study revealed that CHR administration significantly reduced the rise in TNF-α levels in MD-treated rats, suggesting that CHR alleviated the overexpression of inflammatory markers in their ovaries. Concurring with the results of the present investigation, Ai et al. (2013) discovered possible suppression of the pro-inflammatory TNF-α pathway by additional flavonoids.

In the current study, feeding rats MD resulted in a considerable increase in CA-125 levels compared to the control group. CA-125 is a protein produced by various cell types, including ovarian cancer cells (Rao et al., 2021). CA-125 is commonly referred to as a tumor marker or biomarker for ovarian cancer because it provides information about the history of the disease. Measuring CA-125 is the most frequently used test to assist in the diagnosis and follow-up of ovarian cancer (Nossov et al., 2008). According to our findings, the MD-induced increase in CA-125 may indicate a heightened risk of ovarian cancer. However, CHR treatment reduced the elevated CA-125 levels. As per prior reports, oxidative damage and the ROS it produces are thought to be one of the primary initiators of cell death (apoptosis) (Matés et al., 2008; Newsholme et al., 2016; Sifuentes-Franco et al., 2018). The Caspase family of proteases mediates apoptosis, a type of controlled cell death characterized by particular structural alterations (Elmore, 2007). MD treatment caused a state of cell death (Wu et al., 2023). Our findings suggested that marked activation of caspase-3 induced by MD caused inflammation and fibrosis of the ovary. In contrast, CHR treatment inhibited the elevation of caspase-3 level. Thus, the results of the current study suggested that inhibition of caspase-3 activation by CHR results in the prevention of ovarian fibrosis with a significant impact on the production of pro-inflammatory cytokines such as TNF-α and IL-6.

Chemicals that destroy oocytes in primordial follicles might exhibit a delayed effect on the estrous cycle or prolonged reproductive dysfunction, persisting until the recruitment of growing and antral follicles can no longer be sustained (Pocar et al., 2003). Consequently, an increase in ROS in the ovaries leads to fast corpus luteum deterioration, granulosa cell mortality, and a loss of oocyte quality which was present in our histopathological findings in the presence of atretic follicles with degenerated cells and altered corpus luteal size and structure (Peters et al., 2020; Khirallah et al., 2022). Surprisingly, treatment with CHR could restore ovarian tissue damage.

Our research indicates that chrysin, a polyphenol compound with a variety of health-promoting properties, particularly flavonoids, the most prevalent chemical class of phytochemicals, is a promising compound for use in the prevention of ovarian toxicity against toxic agents. In nature, flavonoids can be found everywhere. They can be found in food as well, which makes the connection between nutrition and illness prevention crucial.

## 5 Conclusion

Our study provides novel findings, that exposure to MD in female rats could diminish fertility by inducing oxidative stress, disrupting hormonal balance during reproduction, causing histopathological changes, triggering ovarian inflammation, altering tumor marker levels, and affecting ovarian cell apoptosis. Furthermore, our study illustrated that CHR could mitigate the ovarian damage induced by MD. These findings shed light on the reproductive health risks associated with MD exposure. Further research is needed to fully understand the potential benefits of CHR in preventing MD-induced ovarian damage.

## Data Availability

The original contributions presented in the study are included in the article/supplementary material, further inquiries can be directed to the corresponding author.
